# Transverse Diagnosis and CBCT Technology: A Systematic Review

**DOI:** 10.3390/jcm15020868

**Published:** 2026-01-21

**Authors:** Daniel Diez-Rodrigálvarez, Elena Bonilla-Morente, Alberto-José López-Jiménez

**Affiliations:** 1 Orthodontic Teaching Unit, Mississippi University Institution, 28010 Madrid, Spain; 2 Department of Dental Clinical Specialties, Complutense University of Madrid, 28040 Madrid, Spain

**Keywords:** orthodontics, transverse diagnosis, CBCT, orthopedics

## Abstract

**Background:** Diagnosis is the fundamental basis for understanding biomechanics in orthodontic treatment and for accurately designing the treatment plan. Traditionally, the sagittal plane has been the primary focus of assessment; however, it is essential to consider the patient in all three spatial planes. Therefore, it is necessary to explore the transverse plane, which is equally as crucial as the sagittal and vertical planes. With current technological advances, it is now possible to obtain three-dimensional images of the patient using cone-beam computed tomography (CBCT), allowing evaluation of all planes in a single diagnostic test. This study aimed to assess the diagnostic methods used for transverse analysis and the usefulness of CBCT for this purpose. **Material and Methods**: To select the studies for this review, we searched the PubMed, Scopus, and Cochrane databases for publications between 1965 and 2021. Our inclusion criteria targeted studies that evaluated the transverse plane using CBCT or CT. We assessed the level of evidence according to the OCEBM classification and evaluated the risk of bias using the QUADAS-2 scale. **Results**: After reviewing 535 articles, we selected 16 that met the established criteria. These studies compared various diagnostic methods for transverse analysis and their reproducibility indices. We identified the absence of a gold standard for measuring transverse discrepancies and high variability among diagnostic methods as the main limitations. **Conclusions**: Based on the available evidence, it can be concluded that dental and skeletal transverse discrepancies can be reliably differentiated using the diagnostic techniques evaluated in this study, particularly through CBCT-based assessment. Therefore, the diagnosis of transverse discrepancies should not be considered unclear, as it can be established using objective and measurable criteria. These findings reinforce the clinical value of current diagnostic tools and highlight the importance of accurate three-dimensional interpretation for informed and effective treatment decision-making.

## 1. Background

The success of orthodontics lies in the knowledge of how human beings grow and develop [1]. Diagnosis is fundamental for this knowledge, and it is essential to take into account the three anatomical planes—transverse, sagittal and frontal—so that any malocclusion presented in patients can be treated [2,3].

Traditionally, the sagittal dimension has prevailed, since lateral teleradiography was predominantly used for diagnosis, leaving analysis of the transverse plane an afterthought. Subsequent research has shown that treating the transverse problem first improves sagittal and frontal plane alignment. Thus, we obtained frontal radiographs to evaluate the transverse problem and enhance accuracy [4,5,6,7,8,9].

Furthermore, there are other means to measure the transverse problem. First, there is clinical evaluation by means of study models, whether that be through transverse measurements of intermolar, interpremolar or intercanine widths [10], by evaluating and making a differential diagnosis of the skeletal, dentoalveolar or dental dimension [11], or by measuring the WALA-Ridge [12,13] or the Schwarz vestibulolingual inclinations [14].

Currently, with the introduction of CBCT, intraoral scanners, and other new technologies, obtaining a more accurate transverse diagnosis and identifying the actual cause of the problem is easier [15].

The main objective of treating transverse deficiencies more accurately with CBCT is to improve stability and reduce recurrences, improve smiles, and reduce periodontal problems [15]. Clinicians can meet these objectives by accurately diagnosing and appropriately treating both the skeletal and dentoalveolar dimensions [11].

Thanks to CBCT, diagnostic accuracy is achieved through the study of anatomical structures (midpalatal suture, molar inclination), quantification of the transverse problem, and correct planning of orthodontic treatment [16,17,18,19,20,21].

Therefore, the purpose of this work is to evaluate the scientific evidence and quality of published articles on the use of CBCT for transverse diagnosis and to evaluate the different diagnostic methods for transverse analysis, as well as their replicability.

## 2. Materials and Methods

### 2.1. Protocol and Registration

This systematic review follows the PRISMA (Preferred Reporting Items for Systematic Reviews and Meta-Analyses) checklist, recommended in the American Journal of Orthodontics and Dentofacial Orthopedics [22,23]. The Prisma checklist 2020 is attached as Supplementary Materials.

This study is completed with the PROSPERO registration number (ID: CRD42024512140) [24].

### 2.2. Inclusion and Exclusion Criteria

To ensure the accurate selection of the total number of articles from the different searches and to correctly carry out the systematic review, inclusion and exclusion criteria were established.

The clinical question was generated using the PICO format [25]:

- Population: orthodontic patients in mixed or permanent dentition with erupted first permanent molars.
- Intervention: transverse analysis by means of 3D diagnosis.
- Comparison: study models or frontal radiography, since there is no gold standard.
- Outcome: evaluation of the transverse diagnostic method and replicability of the transverse analysis with 3D diagnostics.
- Study design: prospective and retrospective studies, including randomized clinical trial (RCT), observations and case–control studies.

Excluded:

- Meta-analyses and systematic reviews.
- Publication by eliminating duplicate articles.
- Studies in languages other than English and Spanish.
- Studies on sagittal or frontal dimensions with CBCT only.
- Patients undergoing previous treatments.
- Patients with facial asymmetry; cleft lip and/or craniofacial syndromes.

### 2.3. Sources of Information and Research

We identified the studies through electronic databases: different electronic databases—PubMed, Cochrane and Scopus.

The following search strategy has been carried out using the appropriate descriptors, according to the subject, linked by Boolean operators:

- Transverse analysis AND orthodontics;
- Transverse analysis AND orthodontics AND CBCT;
- Transverse analysis AND orthodontics AND CBCT AND diagnosis;
- CBCT AND diagnosis AND transverse;
- CBCT AND diagnosis AND transverse AND orthodontics.

We recorded the total number of results in a table, organized by database and search strategy (Table 1).

### 2.4. Study Selection and Data Extraction Process

A primary reviewer examined the abstracts and full texts and determined which articles were selected for evaluation and a second reviewer checked for accuracy.

### 2.5. Data List and Level of Evidence

The data collected from each study selected for the systematic review included: author, journal and year of publication, type of study, sample size, study design and main conclusions.

In addition, we assigned each article a level of evidence using the Oxford Centre for Evidence-Based Medicine (CEBM) classification (Table 2) [26].

### 2.6. Risk of Bias in Individual Studies

We used the QUADAS-2 scale to evaluate the quality of the article [40] (Table 3).

## 3. Results

### 3.1. Study Selection

**Figure 1:**
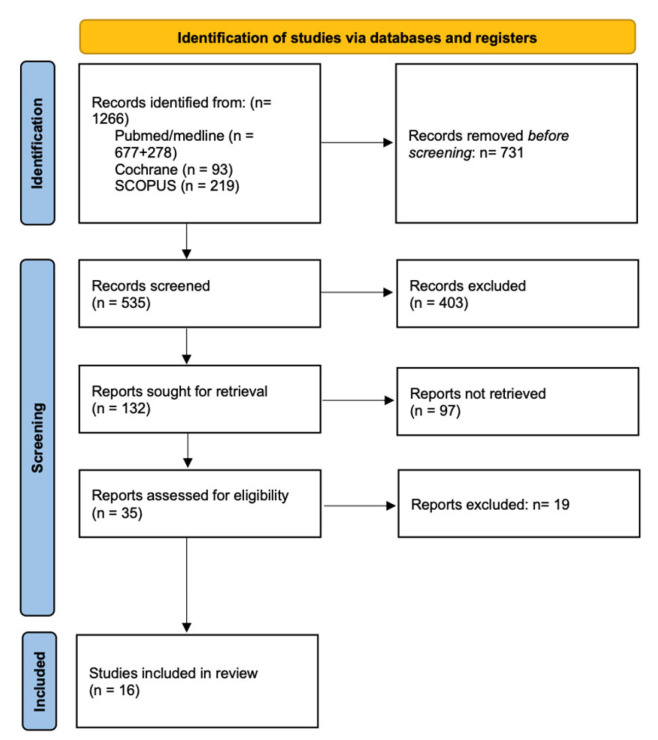
Flow chart.

The flow chart (Figure 1) summarizes the study selection process.

Our initial electronic search across the databases retrieved 1266 articles. We excluded 731 duplicate items and then eliminated 403 articles that did not match the topic, were written in languages other than English or Spanish, or were systematic reviews or meta-analyses. Subsequently, we excluded 97 articles because they contained irrelevant information, lacked an abstract, or involved patients with facial asymmetry, cleft lip, craniofacial syndromes, or previous orthognathic surgery.

After reading 35 articles, 19 articles were discarded due to the lack of scientific evidence, as they were case-related or did not have clear conclusions. Therefore, a total of 16 articles were included, with moderate to high levels of evidence and moderate bias, as shown in the table.

### 3.2. Characteristics of the Study

Table 4 summarizes the key characteristics and results of the studies. All sixteen articles featured English as the primary language and appeared between 2004 and 2020, with sample sizes ranging from 10 to 241 participants. All 16 studies evaluated molar-level dental data, and eight studies also assessed skeletal or dentoalveolar data. Seven studies evaluated the replicability of each analytical tool or technique used to measure or predict transverse changes, while only one reported on specificity and sensitivity (Table 4).

### 3.3. Risk of Bias in the Articles

We evaluated each study’s bias using the QUADAS-2 scale, presenting the specific assessment fields in Table 3.

The studies exhibited heterogeneity and moderate to low methodological quality; consequently, we classified all of them as having a moderate risk of bias. Only one study was of high quality, meeting 65–75%. Eleven studies were of moderate quality, meeting 50–64% of the QUADAS criteria, and four were of low quality. Common weaknesses included inconsistent reference standards due to the lack of a proper gold standard, inadequate sample sizes, lack of blinding, and the use of a spectrum of patients who were not representative of the population that would receive the assessment in practice.

### 3.4. Level of Evidence

The Oxford Centre for Evidence-Based Medicine (CEBM) classification evaluates the level of evidence for each clinical setting [26].

The systematic review has a “moderate level of evidence and grade of recommendation” for transverse analysis using CBCT (Table 2).

## 4. Discussion

Accurate diagnosis represents the cornerstone of successful orthodontic treatment, as it directly influences treatment planning, biomechanics, and long-term stability. Traditionally, orthodontic diagnosis has emphasized the sagittal plane, largely due to the widespread use of lateral cephalometric radiography as the primary diagnostic tool. Consequently, the transverse dimension was frequently underdiagnosed or relegated to a secondary role. However, increasing evidence has demonstrated that transverse discrepancies play a fundamental role in the etiology of malocclusions and that their correction may positively influence sagittal and vertical relationships [9,16,18,20].

The advent of cone-beam computed tomography (CBCT) has profoundly transformed orthodontic diagnostics by enabling three-dimensional visualization of craniofacial structures. This technology allows simultaneous assessment of skeletal, dentoalveolar, and dental components of transverse discrepancies within a single imaging modality [15]. The present systematic review aimed to critically evaluate the scientific evidence regarding the use of CBCT for transverse diagnosis in orthodontics, focusing on diagnostic methods, reproducibility, and clinical applicability [16,17,18,19,20,21].

- *Sample size:*

One of the most relevant findings of this review is the considerable heterogeneity in sample sizes among the included studies. Half of the studies analyzed comprised relatively small samples, ranging from 10 to 29 participants. Such limited sample sizes restrict statistical power and compromise the external validity of the findings, limiting their generalizability to the broader orthodontic population. This limitation is particularly relevant given that transverse discrepancies represent one of the most prevalent orthodontic problems, affecting approximately 48% of patients [9].

In contrast, only three studies included larger samples (121, 133, and 241 participants), allowing more robust statistical analyses and improved extrapolation of results [17,31,33]. These studies provide stronger evidence for normative transverse values and diagnostic thresholds, emphasizing the importance of adequately powered investigations. Future research should prioritize appropriate sample size calculation to ensure reliable and clinically applicable results.

Regarding age distribution, most studies focused on patients between 12 and 16 years of age, which corresponds to the peak period for orthopedic maxillary expansion and orthodontic intervention. This focus is clinically justified, as skeletal responsiveness is greater during adolescence. Nevertheless, only two studies included patients younger than 12 years, and five studies included individuals older than 16 years. This uneven distribution highlights a gap in the literature concerning transverse development in early mixed dentition and transverse diagnostic criteria in adults, in whom skeletal expansion is limited and dentoalveolar compensation is more prevalent.

- *Use of CBCT and software:*

The reviewed studies demonstrated substantial variability in the CBCT devices and software platforms used for image acquisition and analysis. Although iCat scanners V10 and Dolphin software (Imaging 11.9) were the most frequently employed systems, other devices such as NewTom, Galileos, Pax-Zenith 3D, and Scanora 3Dx were also utilized, in combination with diverse analysis software. This heterogeneity reflects differences in institutional availability rather than diagnostic intent.

Importantly, diagnostic accuracy appears to be influenced more by image quality, voxel size, and software measurement precision than by the specific device used. However, the lack of standardized acquisition protocols—including head positioning, volume orientation, and reference plane definition—limits comparability across studies. These inconsistencies reinforce the need for standardized CBCT protocols specifically tailored to transverse orthodontic diagnosis, ensuring reproducibility and minimizing measurement error.

- *Diagnostic method:*

A major finding of this review is the absence of consensus regarding the optimal method for transverse diagnosis using CBCT. Substantial variability exists across skeletal, dentoalveolar, and dental measurement approaches.

### 4.1. Skeletal and Basal Measurements

At the skeletal level, surprisingly few studies evaluated nasal or basal maxillary width. Only two investigations assessed nasal width at the level of the turbinates, limiting conclusions regarding transverse skeletal effects on the nasal cavity [20,39]. Similarly, basal maxillary width assessment was performed only in studies by Podesser et al. and Altieri et al., using the jugal point as a reference [20,39]. The jugal point, defined as the most convex point of the zygomatic alveolar crest, may provide a more accurate representation of basal skeletal width; however, its identification can be challenging due to anatomical variability.

The limited number of studies evaluating true skeletal landmarks underscores a significant gap in the literature. Without consistent skeletal reference points, differentiating skeletal transverse deficiencies from dentoalveolar compensations remains problematic.

### 4.2. Dentoalveolar Measurements

At the dentoalveolar level, marked heterogeneity was observed, particularly in the maxilla. Some studies measured transverse width at the most convex vestibular point of the alveolar bone, analogous to the WALA ridge concept in the mandible [12,13]. Others measured at the palatal aspect, using the curvature of the palatal vault as a reference. These differing approaches yield fundamentally different information and hinder direct comparison.

In contrast, mandibular dentoalveolar assessment was more homogeneous. Most studies used the WALA ridge as a reference landmark, facilitating greater consistency across investigations. This disparity between maxillary and mandibular assessment highlights the need to define standardized maxillary dentoalveolar reference points.

### 4.3. Dental Measurements and Molar Inclination

Dental-level assessment demonstrated greater agreement across studies, particularly regarding intermolar width. Most authors defined maxillary intermolar width as the distance between the palatal cusps of the first molars and mandibular width as the distance between the central fossae. This consistency supports the clinical utility of intermolar width as a reliable dental parameter.

Conversely, molar inclination measurement exhibited substantial methodological variation. Some studies measured inclination relative to the long axis of the tooth and the palatal plane, others used the palatal root axis, and others employed tangential lines from the buccal cusp to the palatal root apex. As a result, reported normative values varied considerably, ranging from approximately 67° to 100° for maxillary molars [21,29,36]. This variability reflects methodological differences rather than true anatomical discrepancies.

In the mandible, most studies measured molar inclination relative to the mandibular plane, but only a few reported normative values [21,36]. The lack of standardized inclination definitions limits the establishment of universally accepted diagnostic thresholds.

- *Replicability index*

The mean data was between 0.85 and 0.98 in the majority of the study. Two of them did not give data, but they did mention that they were replicable. Meanwhile, two studies do not mention a reliability index and only one speaks of it but does not provide data.

After carrying out this review, there is a notable heterogeneity in the diagnosis of the transverse dimension, as well as its quantification. It would be necessary to correctly calculate the sample size in order to have a sufficiently representative sample to ensure the validity and replicability of the method.

At the image and data measurement level, it is necessary to evaluate which is the best CBCT to provide the correct image quality and image sharpness and develop a software that has good accuracy. Thus, this will provide the means to standardize a correct method for transverse problems.

### 4.4. Methodological Quality and Risk of Bias

Assessment using the QUADAS-2 tool revealed that most included studies were of moderate to low methodological quality. Only one study achieved high methodological quality, while the majority exhibited limitations related to small sample sizes, lack of blinding, inconsistent reference standards, and non-representative patient spectra.

These methodological weaknesses necessitate cautious interpretation of the findings. While CBCT offers unparalleled three-dimensional information, the current evidence base does not yet support definitive diagnostic cutoffs or standardized clinical decision-making algorithms.

### 4.5. Clinical Implications of CBCT in Transverse Diagnosis

From a clinical perspective, CBCT provides significant advantages by enabling visualization of skeletal structures, tooth inclination, alveolar bone thickness, and the midpalatal suture. These features allow more precise differentiation between skeletal and dentoalveolar components of transverse discrepancies, which is critical for selecting appropriate treatment modalities.

However, given the moderate level of evidence and concerns regarding radiation exposure, CBCT use should remain selective and indication-based, in accordance with current radiological guidelines [15]. Routine use in all orthodontic patients cannot be justified based on existing evidence.

### 4.6. Future Directions and Research Needs

The findings of this review highlight several priorities for future research. First, a universally accepted gold standard for transverse diagnosis must be established. Second, standardized CBCT acquisition and measurement protocols should be developed, including consistent anatomical landmarks and reference planes. Third, well-designed prospective studies with adequate sample sizes are required to validate diagnostic thresholds and assess their impact on treatment outcomes and stability.

Only through such methodological refinement can the full diagnostic potential of CBCT be translated into predictable and evidence-based clinical practice.

The results of the present study demonstrate that several of the evaluated techniques allow a clear differentiation between dental and skeletal transverse discrepancies. This distinction is clinically relevant, as it directly influences diagnosis, treatment planning, and the selection of appropriate orthodontic or orthopedic approaches. In this context, CBCT has proven to be a reliable diagnostic tool for three-dimensional assessment of the maxillofacial complex, enabling accurate identification of the relative contribution of dentoalveolar and skeletal components to transverse discrepancies. Although additional diagnostic methods may be introduced in the future and considered more practical in specific clinical scenarios, the evidence presented in this study shows that clinicians currently have valid and reproducible tools to accurately diagnose transverse discrepancies and distinguish their underlying components.

## 5. Conclusions

Based on the available evidence, it can be concluded that dental and skeletal transverse discrepancies can be reliably differentiated using the diagnostic techniques evaluated in this study, particularly through CBCT-based assessment. Therefore, the diagnosis of transverse discrepancies should not be considered unclear, as it can be established using objective and measurable criteria. These findings reinforce the clinical value of current diagnostic tools and highlight the importance of accurate three-dimensional interpretation for informed and effective treatment decision-making.

## Figures and Tables

**Table 1:** Search results by database and search strategy used.

	Pubmed	Cochrane	Scopus
Transverse analysis AND orthodontics	623	72	184
Transverse analysis AND orthodontics AND CBCT	495	51	112
Transverse analysis AND orthodontics AND CBCT AND diagnosis	302	49	93
CBCT AND diagnosis AND transverse AND orthodontics	120	23	68
CBCT AND diagnosis AND transverse	544	55	123

**Table 2:** Level of evidence according to OCEMB.

Author	Journal/Year of Publication	Type of Study	Level of Evidence
**Podesser et al.** [20]	European Journal of Orthodontics 2004	Methodological	4
**Domann et al.** [27]	Orthodontics 2011	Retrospective/Ecological	2C
**Shewinvanakitkul et al.** [28]	Orthodontics & Craniofacial Research 2011	Retrospective/Cohort	2B
**Miner et al.** [17]	American Journal of Orthodontics 2012	Retrospective/Ecological	2C
**Akyalcin et al.** [29]	Imaging Science in Dentistry 2013	Retrospective/Ecological	2C
**Woller et al.** [30]	Dental Press Journal of Orthodontics 2014	Retrospective/Cohort	2B
**Miner et al.** [31]	American Journal of Orthodontics 2015	Retrospective/Ecological	2C
**Lin et al.** [32]	The Angle Orthodontist 2015	Retrospective/Cohort	2A
**Yehya Mostafa et al.** [21]	American journal of Orthodontics 2017	Retrospective/Ecological	2C
**Hwang et al.** [33]	American Journal of Orthodontics 2018	Retrospective/Cohort	2B
**Furtado et al.** [34]	Journal of Clinical and Experimental Dentistry 2018	Retrospective/Ecological	2C
**Cantarella et al.** [35]	American Journal of Orthodontics 2018	Retrospective/Ecological	2C
**Golshah et al.** [36]	International Journal of Dentistry	Retrospective/Clinical TRIAL	1B
**Ulema Ribeiro et al.** [37]	International Journal of Paediatric Dentistry 2020	Retrospective/Clinical TRIAL	1A
**Yi et al.** [38]	Sensors 2021	Retrospective/Ecological	2C
**Altieri et al.** [39]	American Journal of Orthodontics 2022	Retrospective/Clinical TRIAL	1A

**Table 3:** Level of risk of bias according to QUADAS-2.

Author	1	2	3	4	5	6	7	8	9	10	11	12	13	14	Total	%
**Podesser et al.** [20]	N	U	Y	N	N	N	Y	Y	N	Y	N	Y	N	N	5.5	39.28
**Domann et al.** [27]	N	Y	N	Y	U	N	N	Y	Y	N	N	Y	N	Y	6.5	46.42
**Shewinvanakitkul et al.** [28]	N	Y	N	N	Y	N	Y	Y	Y	N	N	Y	N	Y	7	50
**Miner et al.** [17]	N	Y	Y	N	Y	Y	N	Y	Y	N	Y	Y	N	Y	8	57.14
**Akyalcin et al.** [29]	N	U	N	Y	U	N	Y	Y	Y	N	N	Y	Y	N	7	50
**Woller et al.** [30]	N	Y	N	Y	N	Y	N	Y	N	N	N	Y	N	N	5	35.71
**Miner et al.** [31]	N	Y	Y	N	Y	Y	N	Y	Y	N	N	Y	N	Y	8	57.14
**Lin et al.** [32]	N	Y	N	Y	N	Y	N	Y	Y	N	N	Y	N	N	6	42.85
**Yehya Mostafa et al.** [21]	N	Y	Y	N	N	N	Y	Y	Y	N	N	Y	Y	Y	8	57.14
**Hwang et al.** [33]	Y	Y	N	N	N	Y	Y	Y	Y	N	N	Y	N	Y	8	57.14
**Furtado et al.** [34]	N	Y	N	Y	N	N	Y	Y	Y	N	N	Y	N	Y	7	50
**Cantarella et al.** [35]	N	Y	Y	Y	Y	U	U	Y	Y	U	U	Y	N	N	9	64.28
**Golshah et al.** [36]	Y	U	Y	U	Y	N	Y	Y	Y	N	N	Y	N	N	7.5	53.57
**Ulema Ribeiro et al.** [37]	N	Y	N	Y	Y	Y	N	Y	Y	N	Y	Y	N	Y	9	64.28
**Yi et al.** [38]	N	U	N	Y	N	N	Y	Y	Y	N	N	Y	Y	Y	7.5	53.57
**Altieri et al.** [39]	Y	Y	N	U	Y	N	Y	Y	Y	Y	N	Y	Y	Y	10.5	75

1–14, Methodologic criteria in Table 2. Y, Yes; fulfilled QUADAS methodologic criteria (1 point). N, No; did not fulfill QUADAS methodologic criteria (0 point). U, Unclear; did not provide sufficient information to evaluate (0.5 point).

**Table 4:** Characteristics of select articles.

Study	Total Number	Average Age	Treatment	CBCT/Software	Measurements	Conclusions
Podeser et al. (2004) [20]	10	26 years & 3 months	Observational (without treatment)	Tomoscan 7000R/Scriptel	- Basal and dentoalveolar widths - Molar inclination	- Reasonable and complementary method for transverse diagnostics - Replicable - Replicability index: 0.48–0.98
Doman et al. (2011) [27]	28	14.1 years	Expansion with Hyrax expander	Sirona Galileos System/Sidexis	- Dentoalveolar effects. - Dental effects: Molar inclination and intermolar distance	- Need to standardize the transverse diagnostic method - Replicability index: 0.9
Shewinvanakitkul et al. (2011) [28]	78	13.2 years	Observational (without treatment)	CB MercuryRay/Accurex by Cybermed	- Lower molar inclination - Lower intermolar distance	- Reasonable method for transverse diagnostics - Replicable - Intermolar distance: 40.9 mm on average - Molar inclination: 74.6° - Replicability index: >0.94
Miner et al. (2012) [31]	241 (with and without crossbite)	13 years	Observational (without treatment)	iCat/Dolphin	- Intermolar-alveolar width - Intermolar distance	- Upper molar inclination 97.77° and 98.29°. - Lower molar inclination: 104.22° and 103.85°. - Replicability index: >0.9
Akyalcin et al. (2013) [29]	24	13.9 years	Hyrax expander	Sirona Galileos System/OsiriX, Pixmeo	- Upper molar inclination - Upper intermolar distance	- Average inclination of 66°–67°. - Replicable - Replicability index: 0.85–0.98
Woller et al. (2014) [30]	25	12.3 years	Hyrax expander	iCat/Dolphin	- Dentoalveolar data - Molar inclination	- Bone changes in all three anatomical planes - Replicability index: 0.947
Miner et al. (2015) [17]	133 (with and without crossbite)	12.8 years	Observational (without treatment)	iCat/Dolphin	- Intermolar-alveolar width - Intermolar distance	- Good predictor of crossbite - Good specificity and sensitivity. - Replicability index: >0.9
Lin et al. (2015) [32]	28	17.4–18.1 years	Hyrax expander and C-Expander	Vatech Implagraphy and Alphad Vega/InVivoDental	- Upper molar inclination - Upper intermolar distance	- Change of 3° inclination after treatment
Yehya Mostafa et al. (2017) [21]	85	10–16 years	Expander and Brackets	CB MercuryRay/Dolphin	- Upper molar inclination 100° - Lower molar inclination 77°	- Improved orthodontic results. - Replicable - Replicability index: 0.98–0.99
Hwang et al. (2018) [33]	121	23–26 years	Observational (without treatment)	Pax-Zenith 3D/OnDemand 3D	- Upper and lower molar inclination. - Intermolar width	- Skeletal changes according to facial growth - Replicable - Replicability index: 0.95
Furtado et al. (2018) [34]	10	12.5 years	Hyrax expander	iCat/OsiriX	- Molar inclination - Intermolar width	- CBCT makes diagnosis more predictable - Replicability Index, but not calculated
Cantarella et al. (2018) [35]	15	17.2 years	Maxillary Skeletal Expander (MSE) with 4 mini-screws	NewTom 5G/OnDemand 3D	- Molar inclination - Intermolar width	- MSE makes expansion more predictable - Reliability index, but not calculated
Golshah et al. 2020 [36]	66	28.74 years	Observational (without treatment)	NewTom 5G/OnDemand 3D	- Upper and lower molar inclination.	- Class I: upper 97°; lower 81° - Class II: upper 92°; lower 84° - Class III: upper 98°; lower 82° - Taking into account the curve of Wilson for standardization - Replicability index: 0.85–0.98
Ulema Riberiro et al. (2020) [37]	29	8.01 years	Hyrax expander	iCat/Dolphin	- Intermolar width - Dentoalveolar data	- Use of CBCT to better quantify. - Rapid maxillary expansion (RME) expands more than slow maxillary expansion (SME), at the skeletal level. - Replicability Index, but not calculated
Yi et al. (2020) [38]	20	8.75–9.09 years	Observational (without treatment)	iCat/Dolphin	- Intermolar width - Intermolar inclination - Dentoalveolar data	- More vertical molar inclination as they grow - Width increases both at the top and at the bottom - Replicability index: 0.91–0.99
Altieri et al. (2022) [39]	26	(G1) = 12.2 ± 0.3 years (G2) = 12.5 ± 0.8 years	(G1): Hyrax expander (G2): MSE with 4 mini-screws	Scanora 3Dx/Simplant	- Intermolar width. - Molar inclination	- Both methods aid in transverse correction - Replicable - Replicability index: 0.85–0.98

## Data Availability

Data sharing is not applicable to this article, as no new data were created or analyzed in this study.

## Supplementary Materials

The following supporting information can be downloaded at: https://www.mdpi.com/article/10.3390/jcm15020868/s1, Table S1: Prisma checklist 2020.
